# Pre-Training Reversible Inactivation of the Basal Amygdala (BA) Disrupts Contextual, but Not Auditory, Fear Conditioning, in Rats

**DOI:** 10.1371/journal.pone.0125489

**Published:** 2015-04-30

**Authors:** Elisa Mari Akagi Jordão, Barbara Kazue Amaral Onishi, Gilberto Fernando Xavier

**Affiliations:** Department of Physiology, Biosciences Institute, University of São Paulo, São Paulo, SP, Brasil; University of Wisconsin-Milwaukee, UNITED STATES

## Abstract

The basolateral amygdala complex (BLA), including the lateral (LA), basal (BA) and accessory basal (AB) nuclei, is involved in acquisition of contextual and auditory fear conditioning. The BA is one of the main targets for hippocampal information, a brain structure critical for contextual learning, which integrates several discrete stimuli into a single configural representation. Congruent with the hodology, selective neurotoxic damage to the BA results in impairments in contextual, but not auditory, fear conditioning, similarly to the behavioral impairments found after hippocampal damage. This study evaluated the effects of muscimol-induced reversible inactivation of the BA during a simultaneous contextual and auditory fear conditioning training on later fear responses to both the context and the tone, tested separately, without muscimol administration. As compared to control rats micro-infused with vehicle, subjects micro-infused with muscimol before training exhibited, during testing without muscimol, significant reduction of freezing responses to the conditioned context, but not to the conditioned tone. Therefore, reversible inactivation of the BA during training impaired contextual, but not auditory fear conditioning, thus confirming and extending similar behavioral observations following selective neurotoxic damage to the BA and, in addition, revealing that this effect is not related to the lack of a functional BA during testing.

## Introduction

Behavioral evidence following either damage or inactivation of the basolateral amygdala complex (BLA) indicates that it is involved in acquisition of both auditory and contextual fear conditioning [1–7]. However, the basolateral complex includes the lateral (LA), the basal (BA) and the accessory basal (AB) nuclei, each of them with distinctive hodology [8]. Electrophysiological studies revealed that while polymodal neurons, i.e., that are responsive to sensory stimuli of different modalities, are more frequently found in the BA, neurons that respond exclusively to stimuli of one sensory modality, i.e., unimodal neurons, are mostly found in the LA [9,10]. Consistent with these data, hodological evidence shows that the hippocampal formation sends major inputs to the BA [9,11–13] and therefore substantially processed information like configurational representations of the environment [14,15]. In contrast, sensory inputs arriving in the LA involve unimodal projections from the thalamus and cortical areas, particularly auditory stimuli [16,17].

Evidence indicates that the LA and BA may differ in their contributions to fear conditioning [15,18–20]. The LA has been the focus of several studies involving auditory fear conditioning [2,21,22]. For instance, pre-training damage to LA prevents acquisition of auditory fear conditioning [2,14,18]. Also, Schafe *et al*. [23] demonstrated that consolidation of auditory fear conditioning is dependent of ERK/MAP Kinase activation in the LA. In addition, while some studies favor interpretations of the LA involvement in contextual fear conditioning [14,24], other studies do not support this conclusion [19,22]. In contrast, it has been claimed that the BA is critically involved with contextual, but not auditory, fear conditioning [15,20].

Contextual fear conditioning requires complex associations of stimuli present in an environment which is then associated with an aversive stimulus. This form of fear conditioning depends on the hippocampus because it requires configural association learning, that is, the conjunct association of several stimuli that then constitute a context [25]. Kim & Fanselow [26] and Anagnostaras *et al*. [27] observed that rats submitted to hippocampal lesions one day after training in a concurrent auditory and contextual fear conditioning task showed deficits in the contextual, but not in the auditory fear conditioning test. Further, Maren *et al*. [28] showed that while post-training dorsal hippocampus NMDA-induced damage severely impaired contextual fear conditioning, no disruption was seen following pre-training damage. In contrast, these authors found that fear conditioning to tone was impaired following both pre- and post-training dorsal hippocampal damage. These data indicate that the hippocampus is required for consolidation of a unified configural representation of the context, but not for a discrete stimulus, and its association with the aversive stimulus [29,30].

Consistent with hodological and electrophysiological evidence, Yaniv *et al*. [15] showed that lidocaine-induced reversible inactivation of the BA during training decreases later freezing responses to the conditioned context, but not to the conditioned auditory stimulus. However, since lidocaine blocks nervous conduction in fibers of passage in addition to decreasing activity in cell bodies [31], and considering the extensive intra-amygdalar projections, these results might not be ascribed exclusively to the inactivation of the BA.

Onishi & Xavier [20] evaluated the effects of pre-training ibotenate-induced damage restricted to the BA on performance of auditory and contextual fear conditioning; the animals were exposed to auditory and contextual fear conditioning simultaneously, and tested separately. The results were straightforward. Damage to the BA disrupted contextual, but not auditory, fear conditioning. An important advantage when using ibotenate-induced BA damage relates to the fact that this procedure avoids damage to the fibers of passage. In contrast, one of the disadvantages of this procedure relates to the fact that it produces a permanent damage thus rendering impossible to specify which memory processes, including acquisition, consolidation, retrieval and/or expression of conditioned responses to the context, were affected.

Reversible inactivation of restricted brain areas in different phases of a memory task has allowed evaluation of their contribution for these different mnemonic processes [32]. Microinfusion of muscimol, a GABA_A_ agonist, has been extensively used to inactivate selected brain areas [4,6,33,34,35]. Unlike lidocaine, muscimol affects only cell bodies leaving fibers of passage functional [36]; thus, it represents an interesting tool for dissecting the contribution of different brain areas for mnemonic processes.

In order to circumvent these limitations, the present study employed muscimol-induced reversible inactivation of the BA to investigate the contribution of this amygdala nucleus for acquisition of auditory and contextual conditioned fear responses, being the retrieval and expression of the conditioned fear tested after the BA inactivation had vanished, therefore with a fully functional BA. In other words, rats submitted to muscimol-induced inactivation of the BA were subjected to concurrent acquisition of auditory and contextual fear conditioning training. Then, after the BA inactivation vanished, the subjects were separately tested in both the auditory and the contextual fear conditioned tasks. Thus, differently from Onishi & Xavier’s [20] study employing selective damage to the BA where retrieval of memory and expression of freezing responses during testing occurred in the absence of a functional BA, in the present experiment the BA was functional during testing.

## Materials and Methods

### Subjects

Sixteen, 3-month-old, naïve male Wistar rats (Biosciences Institute, University of São Paulo) were individually housed in Plexiglas standard animal cages in the animal facility with free access to food and water, under a 12h light: 12h dark cycle (lights on at 0700 h). Temperature was held at 21°C ± 2. All procedures and animal care complied with the guidelines of the Brazilian Society for Neuroscience and Behavior, which conforms to national and international standards and policies, and were approved by the Ethics Committee of the Biosciences Institute at the University of São Paulo (Ref. 2011.1.264.41.8).

### Surgery

The rats were anesthetized with a combination of xylasin (10 mg/kg) and ketamine (100 mg/kg), i.p., and placed in a stereotaxic apparatus (David Kopf Instruments). Four small holes were drilled in the cranium; one overlying the frontal pole for taking the antero-posterior (AP) reference point, one overlying the midline for taking the meso-lateral (ML) reference point, and the remaining two for bilateral implantation of stainless steel guide-cannulae (0.55 mm of external diameter) aiming at 2.0 mm above the BA. Special care was taken to avoid damage to the dura mater. Standard stereotaxic procedures were employed. The stereotaxic coordinates, previously identified in pilot surgeries, followed the Swanson’s [37] rat brain atlas, and were 7.7 mm posterior relative to the frontal pole, 4.8 mm (right) and 5.2 mm (left) middle-lateral relative to the sagittal sinus, and 5.7 mm dorso-ventral relative to the dura mater. Note that there was a medial-lateral asymmetry in the cannulae placements. It has been a common practice in our laboratory to test stereotaxic coordinates for each new lot of rats. These pilot surgeries in a few subjects followed by histological evaluation of the results have allowed refining identification of coordinates. The observed asymmetries seem to occur in rats’ brains of the lineage used in our laboratory.

The guide-cannulae were attached to the skull using dental acrylic anchored by three small screws. In order to avoid obstruction of the cannulae, an obturator was inserted into each of them. After cannulae implantation, the wounds were sutured. Then, the animals received a subcutaneous injection of flunixin (Banamine, 0.215 mg/100g), and were allowed 8 to 10 days of recovery before the beginning of behavioral testing.

### Micro-infusion of muscimol

Rats received bilateral micro-infusion of either 0.16 μL of phosphate buffer saline pH 7.4 (PBS) or muscimol (2 mg/ml) dissolved in PBS, depending on the group, using an infusion-cannula inserted within the guide-cannula. Since the infusion-cannula was 2 mm longer than the guide-cannula, it aimed at the center of the BA. The infusion-cannula (0.3 mm of external diameter) was attached to a micro-syringe (Hamilton, 5 μl) holded on a micro-injector, by way of a PE10 tube. This allowed injection of the specified volume in both hemispheres simultaneously, at the rate of 0.1 μl/min, ten minutes before exposure to the conditioning training.

### Apparati

The auditory and contextual fear conditioning training was conducted in a chamber measuring 30 x 28 x 40 cm, with two black and two white acrylic walls; on the white walls there were conspicuous black geometric forms. The floor chamber is made of 0.3 cm stainless steel rods distant 10 mm from each other (center-to-center); these rods were connected to a shock generator and a scrambler. A homemade computer program activated the shock generator thus releasing footshocks (0.75 mA, 1-s duration) by way of the grid, as scheduled. On three of the walls there were speakers also controlled by the computer program that allowed presentation of 550 Hz tones, at 80 dB-SPL. A video camera installed on the chamber ceiling allowed recording the sessions. This chamber was used both for simultaneous auditory and contextual fear conditioning training and for the contextual fear conditioning testing, when no auditory stimulus was presented.

A distinct cylindrical gray acrylic chamber, measuring 40 cm height and 37 cm in diameter, with a plain plastic floor, also equipped with three speakers thus allowing presentation of 550 Hz tones, at 80 dB-SPL, and a ceiling camera, was used for the auditory fear conditioning testing.

### Behavioral task

Two independent groups of rats received topical injections of either 0.16 μL of phosphate buffered saline (PBS Group, N = 8) or 0.16 μL of muscimol (MUSC Group, N = 8) within the BA nucleus. Ten minutes later these animals were subjected to the simultaneous contextual and auditory fear conditioning training. Each rat was individually placed into the conditioning chamber. After two minutes the animal received five parings of a tone and a footshock (0.75 mA), each pairing presented at 30 s time intervals. The tone lasted 5 s and the footshock lasted 1 s and was concurrent with the last second of the tone.

Twenty-four hours later the animals were subjected to the contextual fear conditioning testing; that is, the rat was placed inside the conditioning chamber along six minutes without any tone and footshock presentations, and the session was video recorded for later measurement of the freezing response. Thirty minutes after the end of the contextual fear conditioning testing, the auditory fear conditioning testing was run. This involved introduction of the subject within the cylindrical gray chamber along two minutes without any stimulus presentation (baseline), followed by presentations of 20 tones (550 Hz, 5-s duration each) at 30-s time intervals; therefore, the auditory fear conditioning testing lasted for 12 minutes. Similarly, behavior was video recorded for later analysis of the freezing response.

### Histology

After the end of the behavioral procedures, all animals received a single 0.16 μL micro-injection of quinolinic acid, a neurotoxic substance, by way of the implanted guide cannulae. This method allows identifying the infusion location and approximately the extent of the BA area reached by the muscimol. Two days after the quinolinic acid injection, the rats were overdosed with sodium pentobarbital (200 mg/kg) and their brains were removed for histological processing. The brains were then sliced in a cryostat (-19°C). Coronal 30-μm-thick sections, taken at every 120 μm at the level of the amygdala were mounted on a glass microscope slide and stained with Cresyl Violet for posterior analysis of the cannulae tracks aiming at the BA, and estimate the extent of the infusion region.

### Data analysis

The time (in seconds) spent exhibiting freezing along the testing sessions, in 1-min time bins, was submitted to repeated measures analysis of variance (ANOVA), having Time Bin as within-subject factor and Drug (either MUSC or PBS) as between-subjects factors. This Time Bin analysis allowed evaluation of the development of the freezing response along the testing session. In the auditory fear conditioning testing, the first and second 1-min duration time bins, without any tone presentation, provided a baseline for the subjects’ freezing response. From the beginning of third time bin onwards, the conditioned 5-s-duration tone was presented at every 30 seconds. Because the tone-induced freezing response lasts longer than the strict period of its presentation, freezing responses were recorded both during the tone presentation and during the time period that followed it. Freezing responses along successive 1-min time bins provided indexes of the fear response both (1) to the tone presentation relative to a corresponding baseline period (time bins 3 and 4 compared to time bins 1 and 2, respectively), and (2) to successive tone presentations along the session (time bins 3 to 12), thus allowing evaluation of the extinction of the fear response to repetitive presentations of the conditioned tone. *Post-Hoc*, Duncan Multiple Range test, was performed when necessary. Differences were considered significant when the P-values were less than 0.05.

## Results

### Histology

Light-microscopic analysis of Nissl-stained brain sections of the MUSC and PBS control rats allowed evaluation of the cannulae tracks and an indirect estimation of the BA region reached by the muscimol, by way of the damage induced by the quinolinic acid infusion after the behavioral testing. Several studies involving topic application of drugs within brain areas use the mechanical damage resulting from the insertion of the infusion cannulae in order to identify the site of injection. However, the location of the particular damage produced by the tip of the infusion cannulae is not always easily identifiable. In the present study we circumvented this problem by infusing quinolinic acid at the injection site and by processing the brains two days later. Because this substance is neurotoxic it induces topic cell loss, thus facilitating later identification of the infusion location in histological slides. Three rats, one of the PBS Group and two of the MUSC Group, were excluded from the behavioral analysis because the infusion-cannulae tracks were not in the aimed location such that the infusion likely reached the anterior LA. The final composition of the groups, then, included seven animals in the PBS group and six animals in the MUSC group.

**Fig 1:**
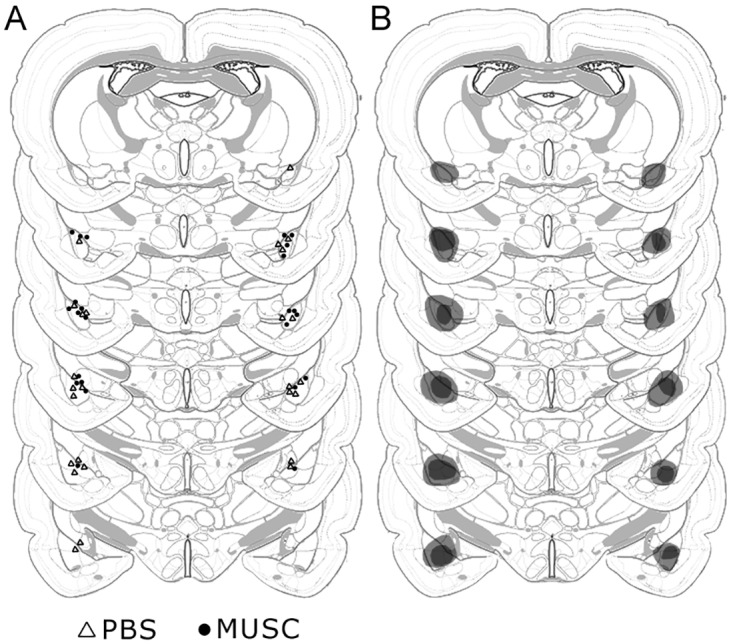
Schematic representations of coronal sections at the level of the amygdala both (A) showing the estimated infusion-cannulae tips placements aimed at the BA, with triangles representing PBS-infused rats (PBS) and circles representing muscimol-infused rats (MUSC), and (B) representing the minimum (black shading) and maximum (gray shading) extent of lesion caused by 0.16 μl of quinolinic acid infused after completion of behavioral testing, thus reflecting approximately the region reached by the muscimol and the PBS infusion, of all subjects which data were included in the behavioral analysis. *Brain images modified from Swanson LW* [38], *author’s copyright*.

**Fig 2:**
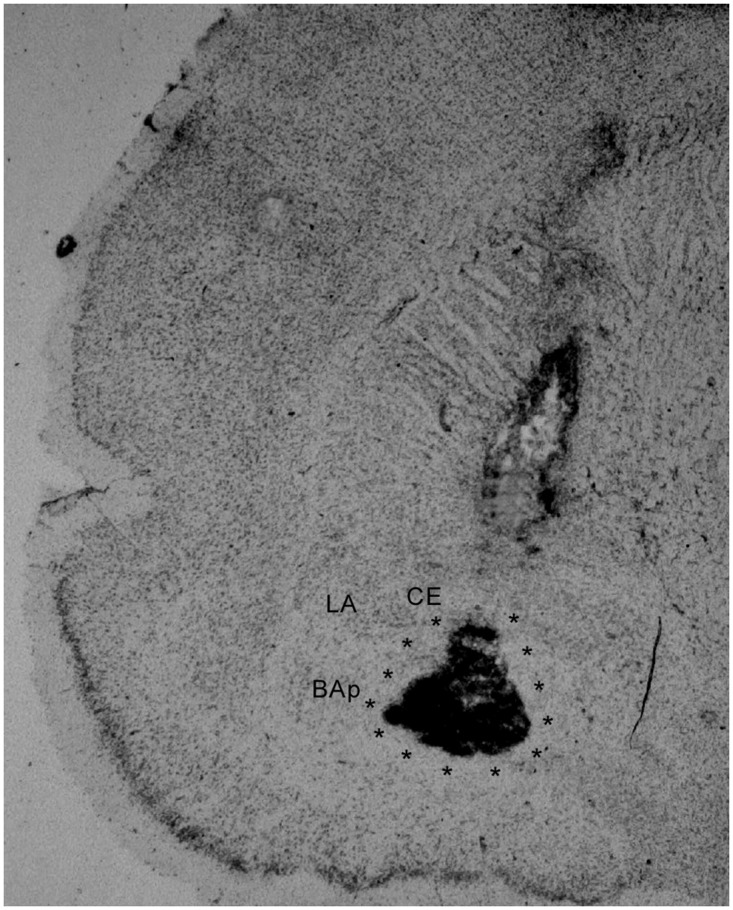
Photomicrograph of cresyl-violet stained coronal brain sections at the level of the BA showing both the lesion caused by the infusion of quinolinic acid after the end of the behavioral testing and the infusion-cannula track on the right hemisphere. Lateral nucleus (LA), posterior basal nucleus (BAp) and central nucleus (CE). Magnification: 40 times.

Fig 1A illustrates in a schematic representation the estimated infusion-cannulae tips placement aiming at the BA in each of the thirteen rats which behavioral data were included in the statistical analysis. Fig 1B illustrates the minimum and maximum damage caused by the infusion of quinolinic acid after the end of the behavioral testing, thus reflecting approximately the infused region, in rats which behavioral data were included in the statistical analysis. This suggests that the muscimol reached mainly the anterior BA and partially (50–60%) the posterior BA. This conclusion is corroborated by the location of the guide-cannulae tracks and infusion-cannulae tips placement, aimed to the anterior part of the BA, in order to avoid muscimol from reaching the LA. Fig 2 shows a photomicrography of cresyl-violet stained coronal brain section of a representative rat. Note both the guide-cannulae and infusion-cannulae tracks reaching the BA, and a selective cell loss restricted to this nucleus.

### Behavior

**Fig 3:**
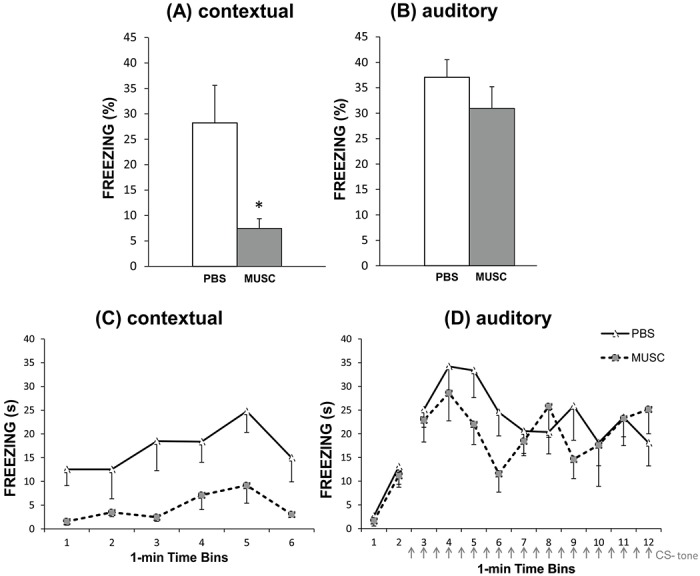
Upper graphics display means (+S.E.M.) of the percentage of time spent exhibiting freezing in the contextual (A) and auditory (B) fear conditioning tests by subjects exposed to either muscimol (MUSC) or phosphate buffered saline (PBS) micro-infusion before conditioning training (*p<0.05). Lower graphics display means (- S.E.M.) of the actual time (in seconds) spent exhibiting freezing along 1-min consecutive Time Bins for subjects micro-injected with either phosphate buffered saline (PBS) or muscimol (MUSC) prior to training, in the contextual (C) and auditory (D) fear conditioning tests. Arrows indicate the moment of the auditory fear conditioning test in which the 5-s-duration CS was presented.

The **percentage of time** the animals spent exhibiting freezing during the fear conditioning test revealed, as expected, that rats infused with muscimol before conditioning training, as compared to PBS injected controls, were disrupted in the contextual (ANOVA, main Drug effect, F_1,11_ = 6.39, p = 0.028) (Fig 3A), but not in the auditory (ANOVA, main Drug effect, F_1,11_ = 0.87, p = 0.37) (Fig 3B), fear conditioning.

The **time** spent exhibiting freezing in 1-min consecutive time bins along the testing sessions is shown in (Fig 3C and 3D). In relation to the evolution of freezing responses during contextual fear conditioning testing (Fig 3C), ANOVA revealed a significant time bin effect (F_5,55_ = 4.73; p < 0.001), with increments of the freezing response along the initial 5 minutes, both for muscimol and PBS injected subjects. These figures suggest that continuous exposure to the conditioned context may facilitate retrieval of the aversive memory for that context, at least in those earlier 5 minutes. Interestingly, this effect was similarly reversed for both groups in the 6th minute, suggesting that extinction started and would have occurred if testing had proceeded for longer. In addition, ANOVA revealed lack of significant Drug x Time Bin interaction effect (F_5,55_ = 0.68, p = 0.64) indicating that these effects equally occurred for both muscimol and PBS injected subjects. Fig 3C also shows that freezing scores of MUSC rats were much lower as compared to those of control rats. *Post-hoc* Duncan’s Multiple Range Test analysis for Drug x Time Bin interaction effect revealed that PBS rats' freezing scores in the fifth Time Bin were greater as compared to Time Bins 1, 2 and 6 (p < 0.05). In contrast, no significant differences were found when comparing MUSC rats' Time Bin freezing scores.

Relative to the evolution of freezing responses during the auditory fear conditioning testing, as Fig 3D shows they were relatively small in the first and second minutes of testing, when no tone was presented. However, they increased dramatically after the tone presentation (starting on the third time bin), gradually decreasing along time bins, then reaching a stable level for both MUSC and PBS rats. Comparisons of freezing responses during the first and second time bins of the auditory conditioning testing, when no tone was presented, with those seen on the third and fourth time bins, when the presentations of the conditioned tone started, revealed (1) a significant main Time Bin effect (F_3,33_ = 32.5; p < 0.0001), (2) lack of main Drug effect (F_1,11_ = 0.51; p = 0.48), and (3) lack of Drug x Time Bin interaction effect (F_3,33_ = 0.19; p = 0.89), thus indicating that freezing reactions of MUSC rats did not differ significantly from those seen in PBS rats, and that both groups exhibited similar reactions to the novel environment. *Post-hoc* Duncan’s Multiple Range Test analysis including these Time Bins revealed a significant increase in freezing scores during the CS presentation as compared to those seen during pre-CS presentation period (p < 0.05). These results reflect, once more, the occurrence of similar auditory fear conditioning in both groups. ANOVA including freezing responses on Time Bins 3 to 12 revealed both no significant main Drug (F_1,11_ = 0.87; p = 0.36) and Time Bin (F_9,99_ = 1.56; p = 0.13) effects, and no significant Drug x Time Bin interaction effect (F_9,99_ = 1.02; p = 0.42), indicating that neither extinction nor retrieval facilitation occurred along testing.

## Discussion

The present study evaluated the effects of pre-training muscimol-induced inactivation of BA on contextual and auditory fear conditioning testing. Results clearly show that rats micro-infused with muscimol before fear conditioning training, as compared to control rats micro-infused with PBS, spent less time freezing during contextual, but not during the auditory fear conditioning test (Fig 3). Thus, reversible inactivation of the BA before a simultaneous contextual and auditory fear conditioning training impaired contextual fear conditioning, leaving auditory fear conditioning intact. This dissociation of performance observed after simultaneous training in a contextual and auditory fear conditioning task, with later tests run separately, have important implications for interpreting the results. That is, generic factors such as reduction of sensitivity to shock or disturbance of attention during the CS-US association after the muscimol micro-injection, that could be invoked to explain conditioning deficits, may all be discarded since acquisition of auditory fear conditioning was completely preserved. This study allows to conclude, now convincingly, that a functional BA is required for acquisition of contextual fear conditioning, in contrast with prior studies which experimental design did not allow this conclusion unambiguously.

Reversible inactivation has been largely employed in behavioral tasks to investigate the role of discrete brain areas in memory processes [4,6,33,34,39]. In contrast to permanent damage that may interfere with several mnemonic processes depending on the moment the damage was induced [32], reversible inactivation of a specific neural region for a relatively known period of time provides a model for evaluating the contribution of that brain area for a given process, without interfering with processes occurring either before or after inactivation. The GABA_A_ agonist muscimol exhibits several advantages in this context. In addition to its strong effect in decreasing firing activity of neurons a few minutes after microinfusion, this effect is restricted to cell bodies leaving fibers of passage intact [40,41].

In this scenario, the present results confirm and extend those reported by Onishi & Xavier [20] showing the effects of selective cell loss in the BA on contextual and auditory fear conditioning. These authors demonstrated that contextual, but not auditory, fear conditioning is impaired after a pre-training ibotenate-induced damage restricted to the BA. However, since this pre-training lesion involves permanent damage to BA before conditioning procedures, rats are trained and tested in the absence of this brain structure, leaving open the possibility that the observed deficit could be related to difficulties either in acquisition or in retrieval of the contextual fear conditioning, or even the expression of fear responses to an aversively conditioned context. In contrast, since the present study employed a pre-training reversible inactivation of the BA, and no muscimol was injected before testing, the BA was inactive during acquisition of fear conditioning, but was functionally recovered for both memory retrieval and expression of fear response during testing to the conditioned context run 24 hours after reversible inactivation. It could be argued that the muscimol-induced inactivation was still present during testing. However, several studies have shown that the duration of the muscimol-induced inactivation is shorter than 24 hours, especially when the injected dose is small [36] as it occurred in the present study. These figures lend support to the view that reversible inactivation of the BA disrupts acquisition of contextual fear conditioning and that its functional recovery before testing does not reverse that disruption.

Similar results were described by Yaniv *et al*. [15] employing pre-training lidocaine inactivation of the BA; that is, there was disruption of contextual, but not auditory, fear conditioning. However, since lidocaine blocks nerve conduction in fibers of passage, one could argue that these behavioral results could be related to lidocaine effects on the fibers passing through the BA, instead of blocking activity of BA neurons. In contrast, since muscimol does not interfere with activity in fibers of passage, only inhibiting neuronal activity, the results of the present study allow to discard the possibility the behavioral impairments observed are related to inactivation of fibers originating in other structures that pass through the BA, thus allowing to conclude that it is related to BA neuronal inhibition.

Reijmers *et al*. [42] used a transgenic mouse (tetTag) to identify activation and reactivation of neurons in amygdala during associative learning and retrieval of aversive memory; a long lasting expression of the gene tau-LacZ (LAC) identified activation of neurons during learning, while a short lasting expression of gene Zif/Egr (ZIF) identified activation of neurons during retrieval. The authors observed a correlation between the amount of freezing during contextual-fear retrieval and the number of reactivated neurons (LAC + ZIF expression) in the BA; in contrast, no correlation was found during auditory-fear retrieval. This evidence implicates activity of BA neurons in both acquisition and retrieval of contextual fear conditioning, but not in auditory fear conditioning. Therefore, it does not conflict with the findings of the present study.

Oliveira Coelho *et al*. [43] showed that the blockade of NMDA receptors in the dorsal hippocampus prior to contextual fear conditioning decreased both CREB (cAMP response element binding) protein phosphorylation ratios (pCREB/CREB) in the LA, BA and CE, and memory consolidation. In addition, these authors also observed higher pCREB/CREB ratios in the BA and CE, but not in the LA, when comparing rats subjected to classical contextual fear conditioning as compared to rats subjected to either immediate-footshock in the conditioning chamber or the conditioning chamber (context) not paired with footshock. Together, these results suggest that the observed increase in pCREB/CREB ratio, required for memory consolidation in the BA is related to the context-footshock association.

The hippocampal formation is responsible for the construction of a conjunctive representation of a context, i.e., it binds together independent features of an environment in one single representation [25,30,44]. This idea has been supported by evidence showing that damage to the hippocampal formation impairs contextual but not auditory fear conditioning [3,26,28]. Hodological studies show that the hippocampal formation targets mainly the BA [11,12]. In addition, electrophysiological evidence shows that neurons located in the BA are mainly polymodal [9]. Furthermore, Yaniv & Richter-Levin [45] and Yaniv *et al*. [46] reported the occurrence of long-term potentiation in the BA following perirhinal and entorhinal cortex stimulation “in vivo”. Together, these evidences lend support to the idea that the BA contributes for context-footshock stimuli association.

Summarizing, in addition to lending support to proposals that the BA is involved in contextual, but not auditory, fear conditioning, thus confirming data of prior studies [15,20], the results of the present study extend them by allowing additional conclusions. That is, since muscimol—induced reversible inactivation of the BA was used, one can discard the possibility that the observed disruption is related both (1) to the blockade of nervous conduction in fibers from other brain structures passing through the BA, as it could be argued relative to the study by Yaniv *et al*. [15] employing lidocaine, and (2) to a dysfunctional BA during testing, as it could be argued relative to Onishi & Xavier’s [20] study involving permanent damage to the BA prior to training, since the subjects of the present study even though trained following BA inactivation, were tested after the BA functional recovery. Therefore, this study adds to our knowledge about the contributions of this amygdalar sub-component, the BA, for fear conditioning. Additional investigation is needed to elucidate the involvement of BA in the distinct processes that occur during acquisition of contextual fear conditioning, including both building a context representation and associating it with the footshock.
